# A novel anti-HER2 monoclonal antibody IAH0968 in HER2-positive heavily pretreated solid tumors: results from a phase Ia/Ib first-in-human, open-label, single center study

**DOI:** 10.3389/fimmu.2024.1481326

**Published:** 2024-11-29

**Authors:** Na Song, Yuee Teng, Jing Shi, Zan Teng, Bo Jin, Jinglei Qu, Lingyun Zhang, Ping Yu, Lei Zhao, Jin Wang, Aodi Li, Linlin Tong, Shujie Jiang, Yang Liu, Liusong Yin, Xiaoling Jiang, Tie Xu, Jian Cui, Xiujuan Qu, Yunpeng Liu

**Affiliations:** ^1^ Department of Medical Oncology, The First Hospital of China Medical University, Shenyang, China; ^2^ Key Laboratory of Anticancer Drugs and Biotherapy of Liaoning Province, The First Hospital of China Medical University, Shenyang, China; ^3^ Department of Clinical Medicine, SUNHO (China) BioPharmaceutical Co., Ltd, Nanjing, China; ^4^ Department of Clinical Medicine, Nanjing Jiening Pharmaceutical Technology Co., Ltd, Nanjing, China

**Keywords:** HER2, IAH0968, clinical study, safety, first-in-human

## Abstract

**Background:**

IAH0968 is an afucosylated anti-epidermal growth factor receptor 2 (HER2) monoclonal antibody which improved the activity of antibody-dependent cellular cytotoxicity (ADCC) and superior anti-tumor efficacy.

**Methods:**

To determine the maximum tolerated dose (MTD) with dose-limiting toxicity (DLT), a single institution, phase Ia/Ib study was undertaken, using 3 + 3 design. The primary endpoints were safety, tolerability and preliminary clinical activity. Eighteen patients were evaluable for safety and fifteen patients were suitable for efficacy analysis. Dose escalations were 6 mg/kg (*N* = 2), 10 mg/kg (*N* = 7), 15 mg/kg (*N* = 5), and tolerable up to 20 mg/kg (*N* = 4).

**Results:**

Only one DLT was found at dosage 10 mg/kg, and no MTD was reached. The most common Grade 3 treatment-related adverse events (TRAEs) were hypokalemia (5.6%), supraventricular tachycardia (5.6%), interval extension of QTC (5.6%), and infusion reaction (5.6%). Grade 4 TRAE was arrhythmia (5.6%). No serious TRAE or Grade 5 was reported. 22.2% of patients had a TRAE leading to dose adjustment and 16.7% of patients had a TRAE resulting in discontinuation of IAH0968. After a median follow-up of 9.7 months (range, 3.7 - 22.0), the objective response rate (ORR) was 13.3% (2/15), the disease control rate (DCR) was 53.3% (8/15), and median progression-free survival (mPFS) was 4.2 months (95% CI: 1.4 - 7.7), and the median duration of disease control (DDC) was 6.3 months (95% CI: 2.9–not reached), with 4/15 responses ongoing.

**Conclusions:**

In HER2-positive heavily pretreated metastatic patients, IAH0968 demonstrated promising clinical activity with durable responses and tolerable safety profiles.

**Clinical trial registration:**

ClinicalTrials.gov, identifier NCT04934514.

## 1 Introduction

Receptor tyrosine-protein kinase erbB-2, also known as HER2, is a member of the EGFR family of receptor tyrosine kinases. Heterodimerization of this receptor with other members of the EGFR family, typically owing to HER2 overexpression, results in the autophosphorylation of tyrosine residues within the cytoplasmic domain of the heterodimer and initiates a variety of signaling pathways leading to cellular proliferation and tumorigenesis (1). The activation of HER2 signaling observed in approximately 20% of breast cancers, and overexpression of HER2 has also been described in a variety of other solid tumors, including gastric and gastroesophageal junction cancers, biliary tract cancer (BTC), colorectal cancer (CRC), non-small-cell lung cancer and bladder cancer, with incidences varying from greater than 50% of uterine cancers to around 2% of non-small-cell lung cancers (1). Compared with other HER-2-negative subtypes, subtypes of HER2-positive cancer share a common mechanism of carcinogenesis characterized by decreased apoptosis and enhanced cell proliferation, mobility, invasiveness, metastasis, and angiogenesis (2). Furthermore, HER2-positive breast cancer is highly immunogenic and aggressive with poor clinical outcomes and high resistance to chemotherapy (3).

HER2-targeted therapies are effective in people with breast cancer and gastric cancer harboring HER2 overexpression and/or amplification. Trastuzumab is the first anti-HER2 monoclonal antibody which is effective in the treatment of breast cancer overexpressing HER2 and is a standard option together with chemotherapy in the treatment of HER2-positive metastatic gastric cancer (4–6). In light of the successes of treatment with trastuzumab and other agents in women with HER2-positive breast cancer, interest has emerged in exploring the efficacy of HER2-targeted therapies. In addition to directly inhibiting tumor cell signaling, antibody-dependent cellular cytotoxicity (ADCC) plays a key role in the mechanism of action of trastuzumab (7). The activity of ADCC can be modulated by the FcγRIIIa polymorphisms expressed on immune effector cells. A number of studies documented a correlation between the objective response rate (ORR) of trastuzumab in humans and their allotype of high-affinity (158 V) or low-affinity (158 F) polymorphic forms of FcγRIIIa (8). Amino acid mutagenesis and glycoform engineering have been used to enhance the binding affinity of Fc fragment to Fcγ receptors to enhance the ADCC activities of antibodies (9–14). ADCC-enhanced anti-HER2 monoclonal antibody is produced by five amino acid mutagenesis and has been approved on by FDA (15). Lack of fucose on human IgG1 oligosaccharide was found to enhance the binding to Fcγ receptor IIIa (FcγRIIIa) and thereby improves (> 50-fold) ADCC (16). Hence, de-fucosylation of trastuzumab can be one of the most promising technologies to improve clinical efficacy and solve the ineffective and resistant of trastuzumab problems. Furthermore, HER2 alterations exist in various other solid tumors, some of which have limited therapeutic options. Therefore, it remains necessary to develop a safe and effective HER2-targeting therapy for the unmet clinical needs.

IAH0968 is an afucosylated anti-HER2 monoclonal antibody which is produced by FUT8-deficient CHO cell line with 100% fucose removal. IAH0968 has shown the same binding properties to HER2 as trastuzumab and enhanced binding affinity to FcγRIIIa allotypes, improved the activity of ADCC and superior anti-tumor efficacy (on-line **Supplementary Table S1**, **Supplementary Figures S1**, **S2**). In cynomolgus monkey studies, IAH0968 showed an excellent safety profile similar to that of trastuzumab. The safety and superior efficacy of IAH0968 supports the clinical studies of IAH0968 in a wider patient population besides breast and gastric cancer, including trastuzumab resistant and ineffective patients.

The present first-in-human study in HER2-positive patients with advanced malignant solid tumors was undertaken to assess the safety, tolerability, PK and preliminary clinical activity of the drug as well as to establish the recommended dose of IAH0968 for Phase II/III trials.

## 2 Methods

### 2.1 Study design and treatment

This was a single institution, phase Ia/Ib study, using 3 + 3 design including dose increase (Phase Ia) and dose expansion (Phase Ib) aiming to determine the maximum tolerated dose (MTD) based on the proportion of patients with dose-limiting toxicities (DLTs) and explore preliminary antitumor activity. This study is registered on ClinicalTrials.gov (NCT04934514). The primary endpoints were safety, tolerability and preliminary clinical activity of IAH0968. Secondary endpoints included pharmacokinetic (PK), recommended phase II dose (RP2D), and anti-drug antibody (ADA). Doses escalation were 6mg/kg (N=2), 10mg/kg (N=7), 15mg/kg (N=5), and tolerable up to 20mg/kg (N=4).

### 2.2 Patient eligibility

Eligible patients with confirmed diagnosis of advanced, unresectable, and/or metastatic HER2 overexpression solid tumor were recruited. Heavily pretreated solid tumors are defined as late stage solid tumor subjects who have failed standard treatment. Standard treatment failure refers to disease progression during or after the last treatment, inability to tolerate toxic side effects during treatment (hematological toxicity ≥ 4 or non-hematological toxicity ≥ 3 after previous standard treatment), disease progression or recurrence during neoadjuvant/adjuvant therapy or within 6 months after the end of treatment. HER2 overexpression was determined by immunohistochemical analysis of tumor tissue as 3+ or 2+ amplification by Fluorescence *in situ* hybridization (FISH). Pertinent eligibility criteria included: age ≥18 years; Eastern Cooperative Oncology Group Performance Status (ECOG PS) of 0/1; recovered from previous systemic therapy-related toxicity; at least one measurable lesion according to Response Evaluation Criteria in Solid Tumors (RECIST) version 1.1; a life expectancy of at least 12 weeks, and adequate organ function.

### 2.3 Assessment

Adverse events (AEs) were assessed and graded by the investigators according to the Common Terminology Criteria for Adverse Events (CTCAE) v5.0. Tumor imaging was performed at baseline, and every two cycles thereafter. Tumor response was assessed by the investigators according to the RECIST, Version 1.1. Antitumor activity assessment was defined as (1) overall response rate (ORR), the proportion of patients who displayed partial response (PR) or complete response (CR); (2) disease control rate (DCR), the proportion of patients who displayed PR or CR or stable disease (SD); (3) progression-free survival (PFS); (4) duration of disease control (DDC); (5) overall survival (OS). DLTs were graded by the CTCAE v5.0 during the MTD evaluation period. MTD was defined as the highest dose with < 25% risk of the true DLT rate > 0.33 during evaluation.

### 2.4 Pharmacokinetics and pharmacodynamics

Blood for single PK analysis was collected at pre-dose, 1 hour post-dose and 0, 2, 4, 8, 12, 24, 48, 72, 120, 168, 240, 312, 384 and 480 hours of the end of the first infusion. Thereafter, samples were collected at pre-dose and 0 hour of the second to fifth infusion. Besides, blood was collected at pre-dose, 1 hour post dose and 0, 2, 4, 8, 12, 24, 48, 72, 120, 168, 240, 312, 384 and 480 hours of the end of the sixth infusion. IAH0968 serum levels were measured with an enzyme-linked immunosorbent assay (ELISA) assay, based on the specific recognition of HER2 by IAH0968. The capture agent was recombinant Human HER2 Protein (Sino Biological, 10004-H02H), coated onto a microplate. Samples were pipetted into the wells and any IAH0968 present will be bound by the coating reagent. After washing away any unbound substances, detection reagent (Biotin-HER2) was added into well to bind with IAH0968. After washing away any unbound detection reagent, streptavidin HRP (Jackson, 016-030-084) was added into well to bind with detection reagent. Following a wash to remove any unbound SA-HRP, a substrate solution was added to the wells and color develops in proportion to the amount of IAH0968 bound in the initial step. The color development was stopped and the intensity of the color was measured. The lower limit of quantification of the assay was 62.5ng/mL. PK parameters were derived from the individual patient serum concentration time profiles after the first and sixth infusion using non-compartmental methods. The maximum (Cmax) and minimum (Cmin) serum concentration after administration were directly taken from analytical data. Dose linearity and proportionality of the PK parameters, Cmax, Cmin, area under the plasma concentration time curve from time 0 to infinity (AUC0–∞) and area under the plasma concentration time curve from time 0 to the last measured concentration (AUC0–last) were investigated over the dose range, based on the individual values by linear regression analysis. A trough level 63.25µg/mL of the drug was set as target for the study, based on previous research that defines 17.75~63.25×10-3µg/mL as the EC50 for anti-proliferative effects and ADCC. Accumulation of IAH0968 was assessed by dividing the Cmax and AUC0~last after the sixth dose by the Cmax and AUC0~last after the first dose.

### 2.5 Statistical analyses

Patients who received at least one dose of IAH0968 were conducted for safety assessments. Three participants withdrew from the study due to infusion reactions and missed the efficacy analysis. The KaplaneMeier method was used to assess PFS and time-to-progression. Cox proportional hazards models were used to estimate the hazard ratio (HR) and 95% confidence intervals (95% CI). All data tests were two-sided testing. Fisher’s exact testing was used to compare the response rate in subgroups. All statistics were analyzed with SAS software (Version 9.4, SAS Institute Inc, Cary, NC).

## 3 Results

### 3.1 Patient characteristics

From July 2021 to November 2022, a total of 18 participants were enrolled into the present trial. Their baseline characteristics are shown in **Table 1**. There were seven males (38.9%) and 11 females (61.1%), with a median age of 52.5 (range 26–70) years. All the participants had an ECOG PS of 1. The primary cancer sites included breast (8/18, 44.4%), colon (3/18, 16.7%), stomach (2/18, 11.1%), rectum (2/18, 11.1%), biliary tract (2/18, 11.1%), and pancreas (1/18, 5.6%). Fourteen patients (77.8%) underwent prior trastuzumab-containing therapy. Five (27.8%) were treated with either one or two lines of therapy and thirteen of whom had three or more lines. All of patients had at least one dose of IAH0968, of whom two patients received 6 mg/kg, seven received 10 mg/kg, five received 15 mg/kg, and four received 20 mg/kg. The median treatment dosage number was two (range 1–21) cycles. The numbers of dosage in different groups are listed in **Table 2**.

**Table 1:** Patient demographics and baseline characteristics.

Characteristic		Total (N=18)
Median age, years (range)		52.5 (26~70)
Sex (%)	Male	7 (38.9)
	Female	11 (61.1)
ECOG performance status (%)	0	0 (0)
	1	18 (100.0)
Primary site (%)	Breast	8 (44.4)
	Colon	3 (16.7)
	Rectum	2 (11.1)
	Stomach	2 (11.1)
	Biliary tract	2 (11.1)
	Pancreas	1 (5.6)
HER2 status (%)	IHC 1+ FISH+	1 (5.6)
	IHC 2+ FISH+	3 (16.7)
	IHC 3+	14 (77.8)
FISH status (%)	Positive	4 (22.2)
	Unknown	14 (77.8)
Metastatic site (%)	Lymph node	10 (55.6)
	Liver	4 (22.2)
	Lung	8 (44.4)
	Bone	2 (11.1)
	Abdominal cavity	4 (22.2)
	Pleura	1 (5.6)
	Brian	1 (5.6)
Number of metastatic sites (%)	1	11 (61.1)
	2	2 (11.1)
	>2	5 (27.8)
Number of lesions (%)	<3	4 (22.2)
	≥3	14 (77.8)
Number of previous lines of therapy (%)	≤2	5 (27.8)
	>3	13 (72.2)
Previous trastuzumab treatment (%)	Yes	14 (77.8)
	No	4 (22.2)

IHC, immunohistochemistry; FISH, ﬂuorescence *in situ* hybridization.

**Table 2:** Dosage numbers in different groups.

		6mg/kg (N=2)	10mg/kg (N=7)	15mg/kg (N=5)	20mg/kg (N=4)	Total (N=18)
Dose number	N (Nmiss)	2(0)	7(0)	5(0)	4(0)	18(0)
	Mean ± SD	1.5 ± 0.71	7.3 ± 7.91	5.8 ± 4.02	2.5 ± 1.29	5.2 ± 5.61
	Median (Q1~Q3)	1.5 (1.0~2.0)	2.0 (2.0~13.0)	8.0 (2.0~8.0)	2.5 (1.5~3.5)	2.0 (2.0~8.0)
	Min~Max	1~2	1~22	1~10	1~4	1~22

### 3.2 Safety analysis

Among all eighteen participants who received at least one dose of IAH0968, 18 (100%) experienced at least one treatment-related adverse event (TRAE), mostly categorized as Grades 1 to 2 (**Table 3**). All four doses were well tolerated. Three patients (16.7%) experienced Grade 3 TRAE, including hypokalemia (5.6%), supraventricular tachycardia (5.6%), QTC interval extension (5.6%), and infusion reaction (5.6%). One subject experienced two TRAE instances at Grade 3. Only one patient (5.6%) experienced Grade 4 TRAE with arrhythmia (10 mg/kg). No serious TRAE or Grade 5 instances were reported, and there were no drug-related deaths. 22.2% (4/18) of patients had a TRAE leading to dose adjustment and 16.7% (3/18) of patients had a TRAE leading to discontinuation of IAH0968. The incidence of infusion reaction was 44.4% (8/18), including two patients with Grade 1, five patients with Grade 2, and one patient with Grade 3. Only one DLT was found at a dosage of 10 mg/kg, and no MTD was reached. The recommended phase-2 dose was set to 10 mg/kg and 15 mg/kg Q3W.

**Table 3:** Most common treatment-related adverse events (any occurred in 10% or more patients and any grade 3/4).

TRAEs	6mg/kg (N=2)			10mg/kg (N=7)			15mg/kg (N=5)			20mg/kg (N=4)			Any (N=18)		
	Grade 3	Grade 4	Any	Grade 3	Grade 4	Any	Grade 3	Grade 4	Any	Grade 3	Grade 4	Any	Grade 3	Grade 4	Any
**Any adverse event (%)**	0	0	2 (100.0)	2 (28.6)	1 (14.3)	7 (100.0)	0	0	5 (100.0)	1 (25)	0	4 (100.0)	3 (16.7)	1 (5.6)	18 (100.0)
Blood and lymphatic system disorders (%)															
Anemia	0	0	0	0	0	5 (71.4)	0	0	5 (100.0)	0	0	2 (50.0)	0	0	12 (66.7)
White blood cell count decreased	0	0	0	0	0	3 (42.9)	0	0	1 (20.0)	0	0	0	0	0	4 (22.2)
Neutrophil cell count decreased	0	0	0	0	0	2 (28.6)	0	0	0	0	0	0	0	0	2 (11.1)
Platelet count decreased	0	0	0	0	0	2 (28.6)	0	0	0	0	0	1 (25.0)	0	0	3 (16.7)
Gastrointestinal disorders (%)															
Diarrhea	0	0	0	0	0	0	0	0	2 (40.0)	0	0	1 (25.0)	0	0	3 (16.7)
Constipation	0	0	0	0	0	0	0	0	0	0	0	0	0	0	0
Liver disorder (%)															
Aspartate aminotransferase increased	0	0	0	0	0	1 (14.3)	0	0	1 (20.0)	0	0	2 (50.0)	0	0	4 (22.2)
Alanine aminotransferase increased	0	0	1 (50.0)	0	0	1 (14.3)	0	0	2 (40.0)	0	0	2 (50.0)	0	0	6 (33.3)
γ-glutamyltransferase increased	0	0	0	0	0	0	0	0	1 (20.0)	0	0	1 (25.0)	0	0	2 (11.1)
Blood bilirubin increased	0	0	1 (50.0)	0	0	0	0	0	0	0	0	0	0	0	1 (5.6)
Cardiac disorder (%)															
Arrhythmia	0	0	0	0	1 (14.3)	1 (14.3)	0	0	1 (20.0)	0	0	0	0	1 (5.6)	2 (11.1)
supraventricular tachycardia	0	0	0	1 (14.3)	0	1 (14.3)	0	0	0	0	0	0	1 (5.6)	0	1 (5.6)
QTC interval extension	0	0	0	1 (14.3)	0	1 (14.3)	0	0	0	0	0	0	1 (5.6)	0	1 (5.6)
Others (%)															
**Infusion reaction (%)**	0	0	1 (50.0)	0	0	3 (42.9)	0	0	3 (60.0)	1 (25.0)	0	2 (50.0)	1 (5.6)	0	9 (50.0)
**Fever (%)**	0	0	0	0	0	1 (14.3)	0	0	1 (20.0)	0	0	0	0	0	2 (11.1)
**hypokalemia (%)**	0	0	2 (100.0)	1 (14.3)	0	1 (14.3)	0	0	0	0	0	0	1 (5.6)	0	3 (16.7)

### 3.3 Efficacy analysis

**Figure 1:**
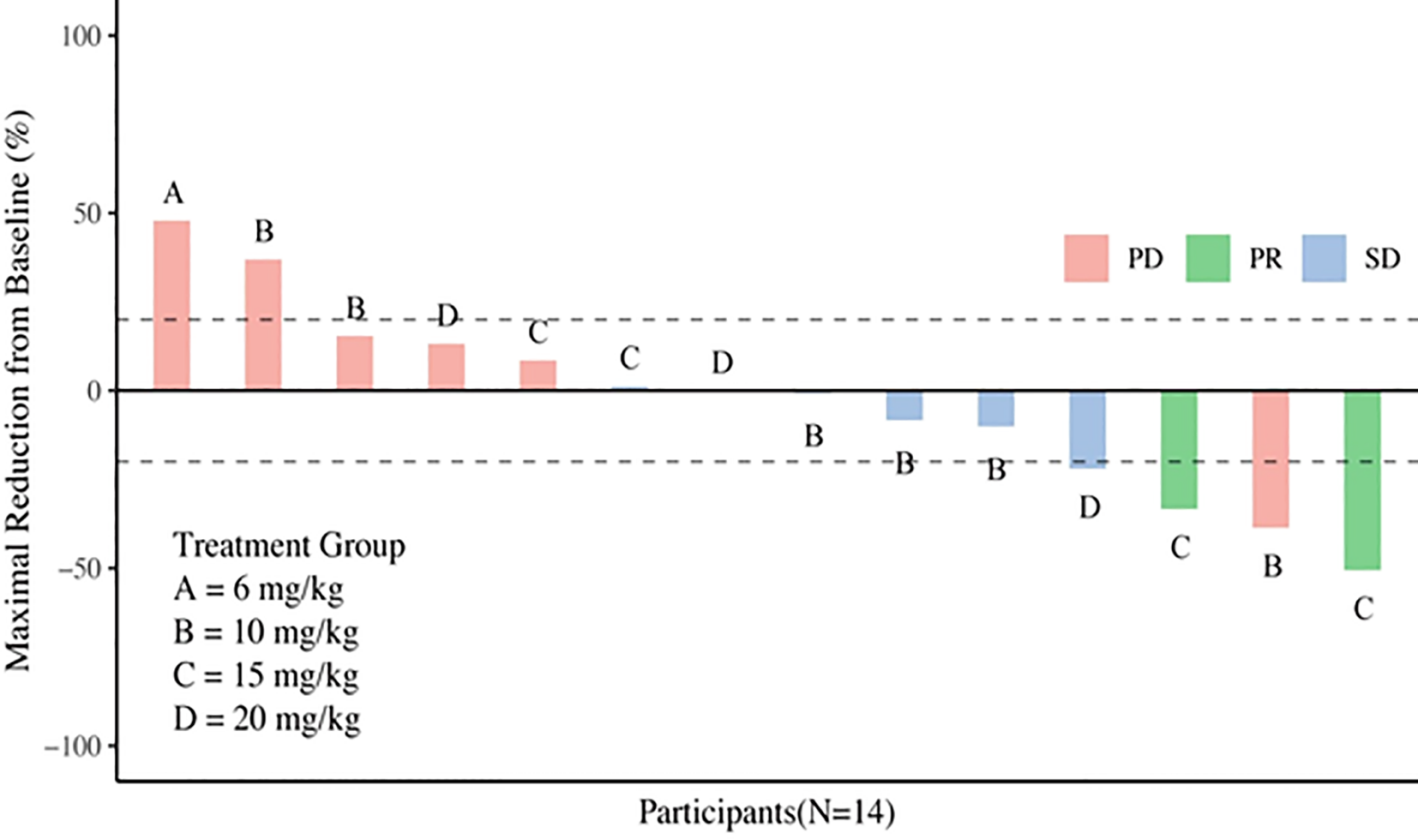
Waterfall plot of best percentage of change assessed by investigator from baseline in sum of diameters in target lesions.

**Figure 2:**
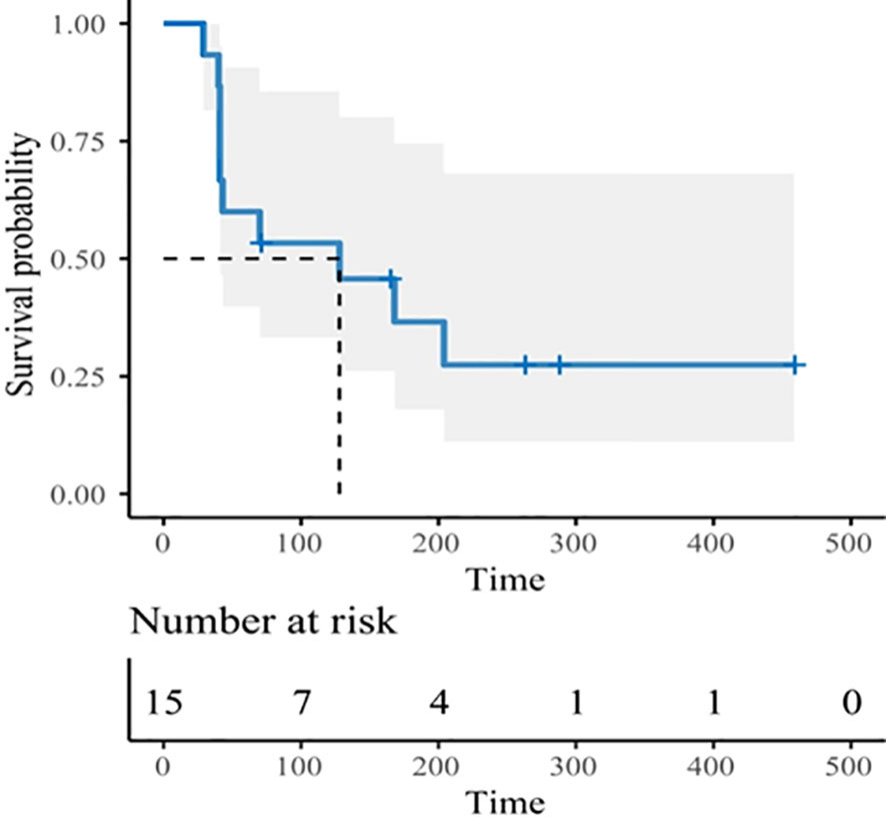
Kaplan–Meier curve for progression-free survival in all participants.

Three subjects withdrew from the study due to infusion reactions and failed efficacy evaluation. In 15 subjects, the ORR was 13.3% (2/15), DCR was 53.3% (8/15) (**Table 4**). Notably, for patients with heavily pretreated HER2+ CRC and BTC (4 received 15 mg/kg and 1 received 10 mg/kg), the ORR was 40% (2/5), and the DCR was 80% (4/5). The best tumor change in sum of diameters for target lesions in all cohorts is shown in **Figure 1** (*N* = 14). With a median follow-up of 9.7 months (range, 3.7 - 22.0) among all eighteen participants, the mPFS was 4.2 months (95% CI: 1.4 - 7.7, **Figure 2**), and mDDC was 6.3 months (95% CI: 2.9–not reached). The OS data are not yet sufficiently mature to report at time of writing. At the time of the data cut-off date of January 15, 2023, there are still four patients remaining on the treatment described herein, and 14 patients discontinued treatment due to disease progression (*N* = 9), TRAEs (*N* = 4), and death (*N* = 1).

**Table 4:** Summary of confirmed responses in 15 participants.

Overall response (%)	6mg/kg (N=1)	10mg/kg (N=7)	15mg/kg (N=4)	20mg/kg (N=3)	Total (N=15)
CR	0	0	0	0	0
PR	0	0	2 (50.0)	0	2 (13.3)
SD	0	3 (42.9)	1 (25.0)	2 (66.7)	6 (40.0)
PD	1 (100.0)	4 (57.1)	1 (25.0)	1 (33.3)	7 (46.7)
ORR (95% CI)					13.3 (1.7-40.5)
DCR (95% CI)					53.3 (26.6-78.7)

Tumor response was confirmed by investigators. ORR includes complete response and partial response. Disease control rates include complete response, partial response, and stable disease ≥12 weeks.

### 3.4 PK evaluation

PK parameters are listed in **Supplementary Table S2**. Mean *T*_1/2_ for doses 6~20 mg/kg IAH0968 ranged from 105 to 118 hours, and CL was from 25.59 to 31.55 mL/h. The volume of distribution (*V*z_-obs_) ranged from 4110.16 mL to 5395.64 mL. A dose for *C*_max_, AUC0~last and AUC0~inf showed a linear increase over the whole IAH0968 dose ranging from 6 to 20 mg/kg. IAH0968 trough level (*C*_min_) by the end of the first cycle was 3.70~14.10 µg/mL from doses ranging from 6 to 20 mg/kg and the EC50 of ADCC activity of IAH0968 was 17.75~63.25 × 10^-3^ µg/mL (**Supplementary Figure S1**). The accumulation factor of IAH0968 was between 0.93 and 1.13, which was assessed by dividing *C*_max_ and AUC0~last after the sixth dose by *C*_max_ and AUC0~last after the first dose. No significant accumulation was observed based on the available data (data not shown).

### 3.5 Immunogenicity evaluation

Samples were screened for ADAs before each infusion of IAH09868 with an electrochemiluminescence assay in bridging format using MSD technology. Briefly, the samples (positive controls, negative controls and study samples) were diluted 1:10 in dilution buffer and treated with 300-mM acetic acid to dissociate complexes of ADA and IAH0968. Then the samples were mixed with Tris-base, added into MSD plates coated by IAH0968, and Ruthenylated-IAH0968 were added to the plate. After 2×MSD read buffer was added and chemiluminescence was measured; the measured light signal was proportional to the concentration of ADA against IAH0968 in the samples. No ADAs were detected at the tested time points except for 1/15 ADA positive at the pre-test (data not shown).

## 4 Discussion

This first-in-human clinical study showed that IAH0968 was well tolerated with a comparable safety profile and encouraging preliminary anti-tumor activity in participants with metastatic HER2-positive solid tumors who had progressed on at least one prior line of anti-HER2 therapy in the metastatic setting. Most AEs experienced were mild or moderate, with only four patients (22.2%) experiencing Grade 3/4 AEs related to IAH0968. Moreover, no Grade 5 AEs occurred. There was no difference in safety among the four dosage groups. The confirmed ORR was 13.3%, and DCR was 53.3%. With a median follow-up of 9.7 months, the mPFS was 4.2 months, and mDDC was 6.3 months.

HER2 is overexpressed in subsets of patients with solid tumors beyond breast cancer and gastric cancer. Therefore, HER2 is an interesting therapeutic target for the development of new drug development, including more potent anti-HER2 antibodies or other methods of targeting HER2. Fc receptor is important for engagement of ADCC against tumor cells, with promising levels of activity reported even among patients with trastuzumab-resistant disease. HER2-targeted antibody-drug conjugate (ADC) and bispecific antibodies are examples of other HER2-targeted agents with potentially more potent anti-tumor activity than trastuzumab. ARX788 is an ADC consisting of HER2-targeted monoclonal antibody conjugated with the AS269 cytotoxic payload, existing promising anti-tumor activity with an ORR of 37.9% and 55.2% DCR (17). Besides, clinical trials with a novel monoclonal antibody margetuximab demonstrated its efficacy in treating HER2-positive breast cancer (18). A humanized, bispecific monoclonal antibody directed against two non-overlapping domains of HER2 named Zanidatamab exerted anti-tumor activity across a range of solid tumors with expression or amplification of HER2 (19). In addition, the HER2 bispecific antibody (KN026) achieved comparable efficacy as trastuzumab and pertuzumab doublet even in the more heavily pretreated patients (20). These Phase 1 data reported here showed that IAH0968 and trastuzumab have comparable PK, safety, tolerance, and immunogenicity. In the present study, all patients were heavily pretreated with a variety of tumor types, and the limited number of patients with heavily advanced disease at the time of enrolment might restrain the therapeutic efficacy. Although all enrolled participates have received anti-HER2 therapies including 77.8% (14/18) with trastuzumab resistance, IAH0968 monotherapy still achieved promising efficacy with an ORR of 13.3% and 53.3% DCR, and an even higher 40% ORR and 80% DCR in patients with heavily pretreated HER2+ CRC and BTC. Therefore, it provides treatment options for patients who have failed to benefit from standard anti-HER2 therapy. These results support that HER2 is an actionable target in various cancer histologies, including BTC and CRC. Considering the limited and heterogenous population in our study cohort, the efficacy is still needed to be validated in future randomized controlled trials for combination treatment.

One of the primary adverse effects of trastuzumab is the increasing risk of cardiac dysfunction, and cardiotoxicity is a typically reversible reduction in left ventricular ejection fraction, and 10.9% patients (19/173) experienced grade 3 cardiac toxicity (21). In this study, IAH0968 monotherapy induced Grade 3/4 cardiac disorders in two patients, including one patient at 10 mg/kg (in the arm) undergoing Grade 3 supraventricular tachycardia and Grade 4 arrhythmia, and the other one at 10 mg/kg (in the arm) developing Grade 3 QTC interval extension. None of the participates experienced reduction in left ventricular ejection fraction. Only one DLT was found at a dosage of 10 mg/kg, and no MTD was reached. Finally, the IAH0968 dose reached 20 mg/kg with sustained anti-tumor activity.

While various anti-HER2-directed therapies have shown variable success in a large set of HER2-positive carcinomas, they always face the problems of drug resistance including both primary and acquired resistance. Genetic aberrations in downstream signaling and genomic alterations beyond ERBB2 might compensate for HER2 suppression (22, 23). In addition, loss of HER2 expression after failure of trastuzumab-containing chemotherapy concerns for one source of acquired resistance mechanisms (24). Therefore, reassessing the HER2 status following first-line trastuzumab before second-line trials is necessary by repeat biopsy or ctDNA. This study did not evaluate the HER2 status before IAH0968 monotherapy. Therefore, whether the absence of HER2 status affects the efficacy of IAH0968 therapy remains unclear. In summary, a deeper understanding of the molecular environment will lead to a greater understanding of the mechanisms of to HER2-directed therapies, and further evidence of combining approaches to target compensatory pathways with anti-HER2 agents is worth exploring. Besides, more translational research is required to reveal the promising biomarkers in predicting better response to HER2-targeted agents.

There are three limitations in this study. The major limitation is the small sample size. The second limitation is the HER2 status was not re-evaluated. Furthermore, the study design omits collection of blood and tissue samples for exploratory analysis.

In conclusion, IAH0968 monotherapy demonstrated tolerable safety and promising anti-tumor activity with a confirmed ORR of 13.3% and DCR of 53.3%. Notably, for patients with heavily pretreated HER2+ CRC and BTC, the ORR was 40%, and the DCR was 80%. Furthermore, the mPFS was 4.2 months, and mDDC was 6.3 months. Based on these encouraging results, a randomized controlled, open-label phase II/III study to evaluate the efficacy of IAH0968 combined with chemotherapy as first-line treatment with HER2-positive advanced colorectal cancer is ongoing (Chinadrugtrials.org.cn: NCT05991518).

## Data Availability

The original contributions presented in the study are included in the article/**Supplementary Material**. Further inquiries can be directed to the corresponding authors.

## Supplementary material

Supplementary Figure S1 Enhanced ADCC activity of IAH0968 with two FcγRIIIa allotypes. The NK92-MI-CD16a (158 V/V allele) and NK92-MI-CD16a (158F/V allele) cells were used as effector cells and HER2-expressed BT474 cell was used as target cell. The ratio of effector cells and target cell (E:T) was 5:1. The specific lysis was evaluated using an LDH release assay (CytoTox 96 Non-Radioactive Cytotoxicity Assay; Promega) according to the manufacturer’s protocol. The percentage of specific lysis was calculated: 100 x (experimental release -spontaneous release)/(maximum release - spontaneous release).

Supplementary Figure S2 Superior *in vivo* efficacy of IAH0968 compared with trastuzumab. Established human breast cancer cells BT474 xenografts in female BALB/c nude mice were treated with vehicle (black), 5mg/kg trastuzumab(green) and three doses of IAH0968, 1mg/kg, 5mg/kg, and 30mg/kg by intraperitoneal administration biweekly for 4 weeks. Dosing was started at the average tumor size of approximately 190 mm^3^. Tumor volume was measured for each group.
