# Supplementary figures and images for: A novel anti-HER2 monoclonal antibody IAH0968 in HER2-positive heavily pretreated solid tumors: results from a phase Ia/Ib first-in-human, open-label, single center study
Source: Front Immunol. 2024 Nov 29;15:1481326. doi: 10.3389/fimmu.2024.1481326 (PMC11637859; doi:10.3389/fimmu.2024.1481326)

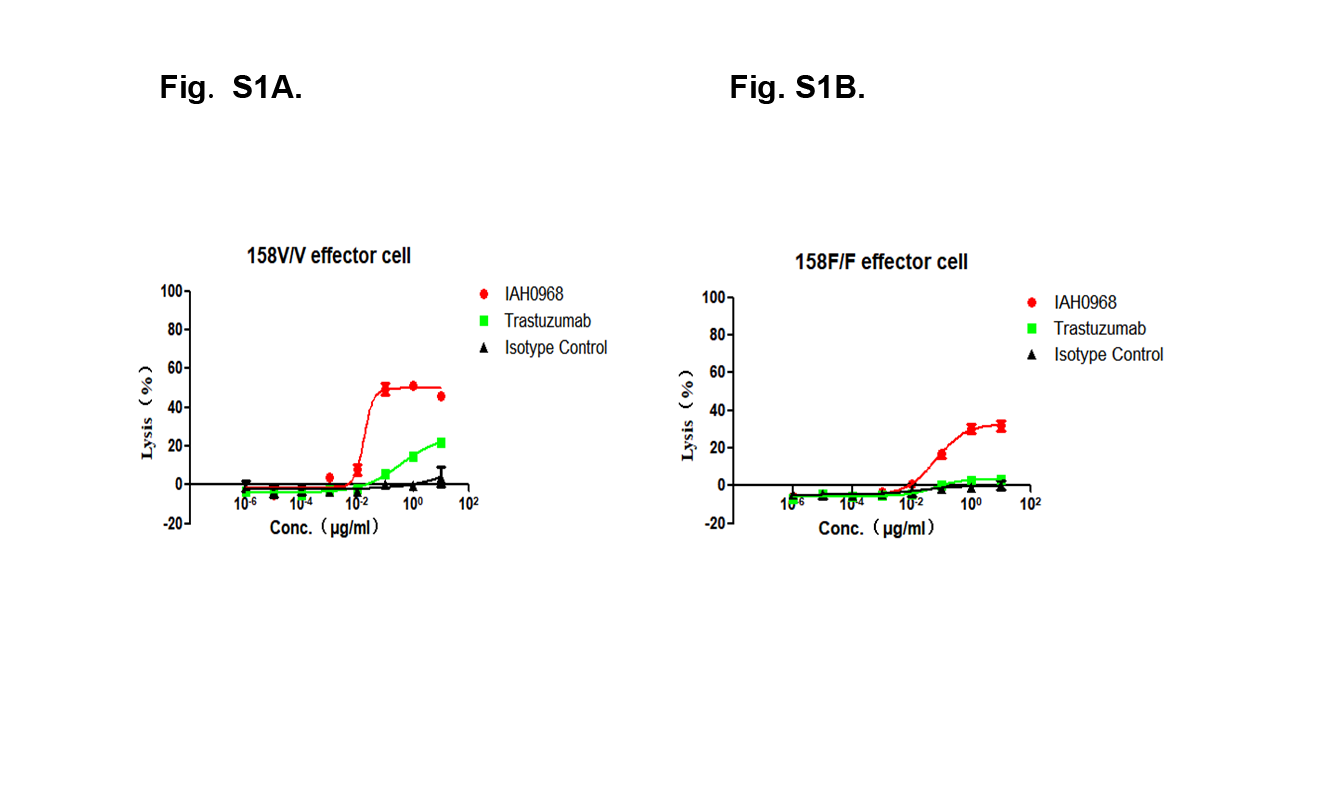

Supplement: Supplementary Figure S1 — Enhanced ADCC activity of IAH0968 with two FcγRIIIa allotypes. The NK92-MI-CD16a (158 V/V allele) and NK92-MI-CD16a (158F/V allele) cells were used as effector cells and HER2-expressed BT474 cell was used as target cell. The ratio of effector cells and target cell (E:T) was 5:1. The specific lysis was evaluated using an LDH release assay (CytoTox 96 Non-Radioactive Cytotoxicity Assay; Promega) according to the manufacturer’s protocol. The percentage of specific lysis was calculated: 100 x (experimental release -spontaneous release)/(maximum release - spontaneous release). [file Image1.tif]

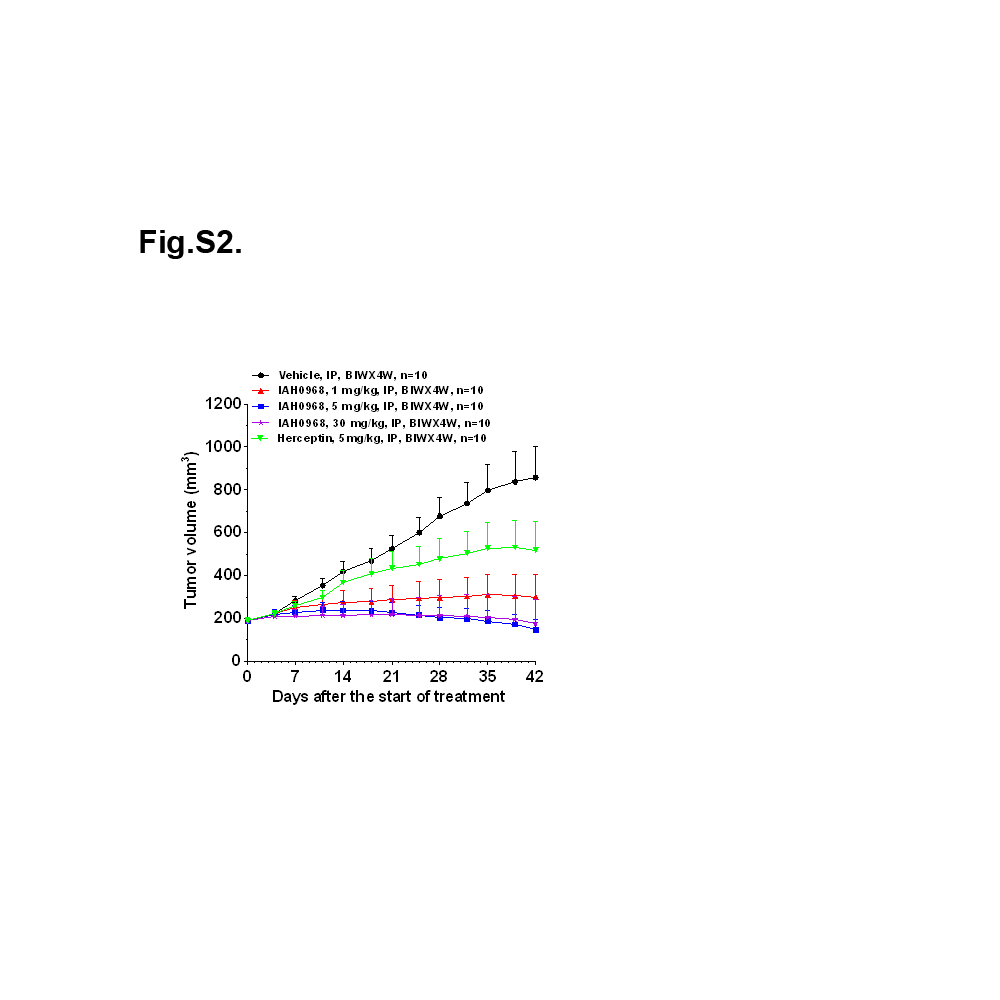

Supplement: Supplementary Figure S2 — Superior in vivo efficacy of IAH0968 compared with trastuzumab. Established human breast cancer cells BT474 xenografts in female BALB/c nude mice were treated with vehicle (black), 5mg/kg trastuzumab(green) and three doses of IAH0968, 1mg/kg, 5mg/kg, and 30mg/kg by intraperitoneal administration biweekly for 4 weeks. Dosing was started at the average tumor size of approximately 190 mm3. Tumor volume was measured for each group. [file Image2.tif]
