# Supplementary material for: A novel anti-HER2 monoclonal antibody IAH0968 in HER2-positive heavily pretreated solid tumors: results from a phase Ia/Ib first-in-human, open-label, single center study
Source: Front Immunol. 2024 Nov 29;15:1481326. doi: 10.3389/fimmu.2024.1481326 (PMC11637859; doi:10.3389/fimmu.2024.1481326)
Supplement: Supplementary file 3 [file Table1.docx]

## Supplementary Material

## Table S1. Enhanced Binding affinity to FcγRIIIa allotypes

| FcγRs | IAH0968 | Trastuzumab |
| --- | --- | --- |
| FcγRIIIa（158V/V） | 25.9 nM | 275 nM |
| FcγRIIIa （158F/F） | 79.3 nM | 1560 nM |

The binding affinity to FcγRIIIa allotypes was measured using BLI (Biolayer interferometry, Octet RED96e, ForteBio). The FcγRIIIa allotypes was bounded on the sensor biolayer, then series diluted IAH0968 or Trastuzumab samples was applicated, and finally the binding reached an equilibrium state. The intermolecular interaction process can be recorded in real time, for the determination of association rate (Kon), dissociation rate (Kdis), affinity (KD).

Table S2 Pharmacokinetic parameters of single intravenous infusion injection of IAH0968 in Phase 1 clinical trial

| Dose  (mg/kg) | N  patients | Cmax  (ng/mL) | Cmin  (ng/mL) | AUC0~last  （h*ng/mL） | AUC0~inf  （h*ng/mL） | T_1/2_（h） | Vz-obs  （mL） | CL  （mL/h） |
| --- | --- | --- | --- | --- | --- | --- | --- | --- |
| 6 | 1 | 154383 | 3702.7 | 14496393 | 15059300 | 105 | 4581.13 | 30.49 |
| 10 | 7 | 195453±21578 | 6800.6±4240.7 | 18232002±3736704 | 20162139±3602857 | 110±32.5 | 4709.19±1020 | 31.26±7.51 |
| 15 | 4 | 360039±61452 | 11529.6±6301.8 | 35733883±3545916 | 37794109±4122815 | 110±36.5 | 4110.16±2100 | 25.59±7.2 |
| 20 | 3 | 393713±120419 | 14096±2318.7 | 37920327±8941578 | 40335413±9235326 | 118±6.44 | 5395.64±456 | 31.55±1.46 |
